# Rescue of high-specificity Cas9 variants using sgRNAs with matched 5’ nucleotides

**DOI:** 10.1186/s13059-017-1355-3

**Published:** 2017-11-15

**Authors:** Sojung Kim, Taegeun Bae, Jaewoong Hwang, Jin-Soo Kim

**Affiliations:** 1 Center for Genome Engineering, Institute for Basic Science, Seoul, South Korea; 2 IBS School, Korea University of Science and Technology, Seoul, South Korea; 3 Department of Chemistry, Seoul National University, Seoul, South Korea

**Keywords:** CRISPR-Cas, Off-target effect, Engineered Cas9 variants, Hammerhead ribozyme-linked sgRNA

## Abstract

We report that engineered Cas9 variants with improved specificity—eCas9-1.1 and Cas9-HF1—are often poorly active in human cells, when complexed with single guide RNAs (sgRNAs) with a mismatch at the 5’ terminus, relative to target DNA sequences. Because the nucleotide at the 5’ end of sgRNAs, expressed under the control of the commonly-used U6 promoter, is fixed to a guanine, these attenuated Cas9 variants are not useful at many target sites. By using sgRNAs with matched 5’ nucleotides, produced by linking them to a self-cleaving ribozyme, the editing activity of Cas9 variants can be rescued without sacrificing high specificity.

**Electronic supplementary material:**

The online version of this article (doi:10.1186/s13059-017-1355-3) contains supplementary material, which is available to authorized users.

## Background

Clustered, regularly interspaced, short palindromic repeats (CRISPR) – CRISPR-associated (Cas) RNA-guided endonucleases, derived from adaptive immune systems in bacteria and archaea, have been repurposed for targeted genome editing in various cells and organisms [1–4]. These nucleases cleave chromosomal DNA in a targeted manner, producing site-specific DNA double-strand breaks (DSBs), the repair of which via non-homologous end-joining (NHEJ) induces small insertions or deletions (indels) at target sites. Unfortunately, off-target DNA cleavage at sites that are highly homologous to on-target sites can lead to mutations at undesired genomic loci [5, 6] and to chromosomal rearrangements such as translocations and inversions [6–8]. Both *S. pyogenes* Cas9 [9, 10] and single guide RNAS (sgRNAs) [6, 11, 12] have been modified to minimize or eliminate these off-target effects. In particular, two groups have independently presented engineered Cas9 variants, termed enhanced Cas9-1.1 (eCas9-1.1) [9] and Cas9 high-fidelity variant 1 (Cas9-HF1) [10], with minimal or no detectable off-target effects in human cells. These high-specificity Cas9 variants contain alanine substitutions to weaken non-specific ionic interactions between the Cas9 protein and the non-target or target DNA strand.

Here, we show that these attenuated Cas9 variants are poorly active at sites with a mismatched 5’ nucleotide relative to their sgRNA sequences in human cells. By using sgRNAs with matched 5’ nucleotides relative to their target DNA sequences, generated by self-cleaving ribozyme fusion, the cleavage activity of the Cas9 variants was rescued in human cells without sacrificing their high specificities.

## Results

**Fig. 1:**
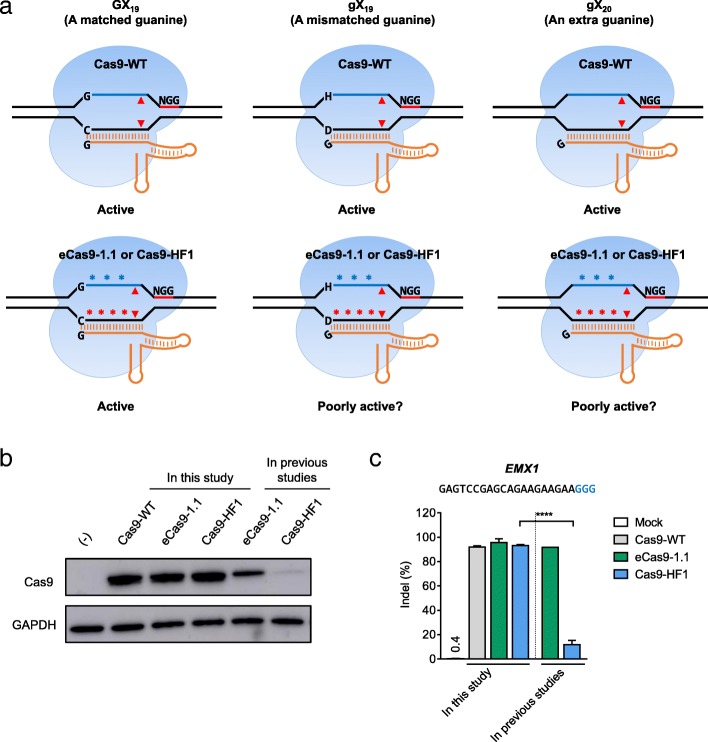
High-specificity Cas9 variants with attenuated cleavage activity. **a** *Schematics* of Cas9-WT, engineered Cas9 variants, and sgRNA variants. Locations of the alanine substitutions introduced in Cas9-WT to create eCas9-1.1 or Cas9-HF1 are shown with *blue* or *red asterisks*, respectively. *Red triangles* indicate cleavage positions. GX_19_ sgRNA starts with a G matched to its protospacer (*blue line*). gX_19_ sgRNA has a mismatched G at its 5’-end. gX_20_ sgRNA includes an extra guanine at its 5’ terminus. The protospacer-adjacent motif (PAM) is shown by a *red line* labeled NGG. H, not G (A or C or T); D, not C (A or G or T). **b**, **c** *Western blot* (**b**) and indel frequencies at the *EMX1* site (**c**) of HeLa cells co-transfected with plasmids encoding Cas9-WT or variants and *EMX1*-specific sgRNA. Cas9 variants were expressed by new plasmids used in this study or by old plasmids used in previous studies. Error bars, s.e.m of two or three biological replicates

We hypothesized that the attenuated Cas9 variants might be poorly active at sites with a mismatch at the 5’ terminus. Because the U6 promoter, which is commonly used to express sgRNAs in eukaryotic cells, requires a guanosine (G) nucleotide to initiate transcription, sgRNAs typically contain a G nucleotide at the 5’ terminus. Three out of four DNA target sites will contain a mismatch at this position and thus might be poorly edited in cells by attenuated Cas9 variants in complex with gX_19_ sgRNAs (Fig. 1a), where “g” or “G” is a mismatched or matched guanosine, respectively. Note that high-specificity Cas9 variants have been previously tested at target sites with a G nucleotide at the 5’ end of the target DNA strand using GX_19_ sgRNAs [9, 10].

Before testing this hypothesis, we compared expression levels of Cas9 variants in human cells and found that the two variants, especially Cas9-HF1, were poorly expressed in HeLa cells (Fig. 1b). We noted that our plasmid encoding wild-type Cas9 (termed Cas9-WT hereinafter) and the two plasmids encoding Cas9 variants [9, 10] contained different promoters, tags, and codon sequences [4, 6]. We performed site-directed mutagenesis in our Cas9-WT plasmid to obtain constructs encoding the two high-specificity Cas9 variants. Western blot analysis showed that all three proteins were highly expressed in HeLa cells (Fig. 1b). Consistent with this result, eCas9-1.1 and Cas9-HF1 expressed using the new constructs induced indels at the *EMX1* site with efficiencies comparable to Cas9-WT (Fig. 1c). Based on these results, we used the newly cloned constructs to express eCas9-1.1 and Cas9-HF1 throughout this study.

### Reduced editing activity of high-fidelity Cas9 variants at target sites with a mismatched 5’ nucleotide

**Fig. 2:**
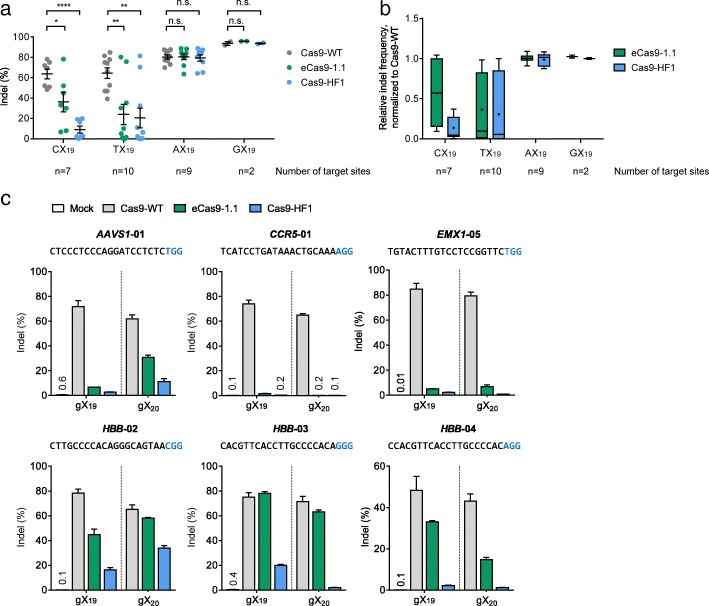
Reduced editing activity of high-fidelity Cas9 variants at target sites with a non-G 5’ nucleotide. **a** Indel frequencies of Cas9-WT and Cas9 variants at 26 endogenous target sites with an HX_19_ sequence obtained using gX_19_ sgRNAs in HeLa cells (H: not G [C or T or A]). The sequences and indel frequencies (%) of the 26 target sites are described in Additional file 1: Table S1. * *P* < 0.05, ** *P* < 0.01, **** *P* < 0.0001. **b** Distributions of relative indel frequencies of eCas9-1.1 and Cas9-HF1 normalized to that of Cas9-WT. Means and medians of relative indel frequencies are represented in Additional file 1: Table S2. *Box and whisker plots*: *center lines* show the medians; *crosses* show the means. **c** Indel frequencies (measured by targeted deep sequencing) at six on-target sites with a 5’ C or 5’ T nucleotide obtained using gX_19_ and gX_20_ sgRNAs in HeLa cells. PAM sequences are shown in *blue*. Error bars, s.e.m. of three biological replicates

To test whether the attenuated Cas9 variants are poorly active at sites with a mismatch at the 5’ terminus, we compared editing activities of eCas9-1.1 and Cas9-HF1 with those of Cas9-WT at 26 sites whose 5’ terminal nucleotides are not guanosine in HeLa cells using gX_19_ sgRNAs: the sites with a 5’ cytosine (C) were termed CX_19_ (seven sites); those with a 5’ thymine (T), TX_19_ (ten sites); and those with a 5’ adenosine (A), AX_19_ (nine sites) (Fig. 2a and b, Additional file 1: Table S1 and Table S2). As expected, Cas9-WT was not sensitive to the mismatch at the 5’ end, inducing indels at high frequencies (64 ± 5% at CX_19_ sites; 65 ± 5% at TX_19_ sites; 80 ± 2% at AX_19_ sites, on average). eCas9-1.1 showed much lower indel frequencies at CX_19_ sites (36 ± 10%) and TX_19_ sites (24 ± 10%), a 1.8-fold or 2.7-fold reduction in average indel frequencies at CX_19_ or TX_19_ sites, respectively. Cas9-HF1 was least active among the three Cas9 nucleases, with average indel frequencies of 9.0 ± 3% at CX_19_ sites and 20 ± 10% at TX_19_ sites, which corresponds to 7.1-fold and 3.2-fold reductions, respectively. At AX_19_ target sites, however, both eCas9-1.1 and Cas9-HF1 showed indel efficiencies (81 ± 3% and 79 ± 3%) comparable to that of Cas9-WT (80 ± 2%), suggesting that a G:T mismatch at the 5’ terminus may still form a wobble base pair. These results are in line with a previous report showing Cas9-HF1 activities with three and one gX_19_ sgRNAs at CX_19_ and AX_19_ sites, respectively [10].

We chose six CX_19_ or TX_19_ target sites at which the two Cas9 variants were poorly active and tested gX_20_ sgRNAs with an extra guanosine at the 5’ terminus rather than gX_19_ sgRNAs (Fig. 2c). Note that gX_20_ sgRNAs, unlike gX_19_ sgRNAs, have matched nucleotides at the 5’ end. Use of gX_20_ sgRNAs enhanced the activity of Cas9 variants at *AAVS1*-01 and *HBB*-02 sites but reduced the activity at the other four sites, compared to gX_19_ sgRNAs. We also noted that Cas9-WT was more efficient with gX_19_ sgRNAs than with gX_20_ sgRNAs at all six sites. These results show that gX_20_ sgRNAs cannot rescue the genome editing activities of high-specificity Cas9 variants. These Cas9 variants in combination with gX_19_ and gX_20_ sgRNAs also showed lower indel frequencies than Cas9-WT in HEK293T, another human cell line (Additional file 1: Figure S1).

### Rescue of high-specificity Cas9 variants using Hammerhead ribozyme-linked sgRNAs

**Fig. 3:**
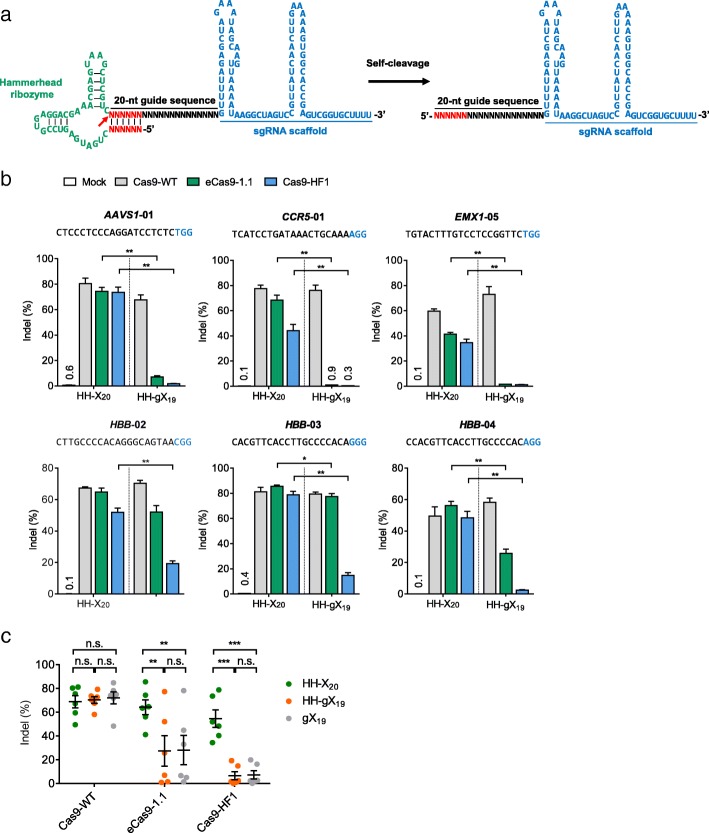
Recovery of editing efficiency of high-fidelity Cas9 variants using HH ribozyme-linked sgRNAs. **a** A *schematic* of a self-processing ribozyme fused sgRNA. The pre-sgRNA contains a HH ribozyme at its 5’-end. The pre-sgRNA undergoes self-cleavage to release a mature sgRNA. The *red arrow* indicates the self-cleavage position. **b** HH ribozyme-fused sgRNAs with a matched 5’ nucleotide (HH-X_20_) or a mismatched guanosine (HH-gX_19_) were tested in combination with Cas9-WT and high-fidelity Cas9 variants at six target sites in HeLa cells. Indel frequencies were measured using targeted deep sequencing. The PAM is shown in *blue*. Error bars, s.e.m. of three biological replicates. Statistical significances were calculated by unpaired t-test. * *P* < 0.05, ** *P* < 0.01. **c** Mean indel frequencies ± s.e.m. at the six target sites in HeLa cells. Statistical significances were calculated by paired t-test. ** *P* < 0.01, *** *P* < 0.001

To expand the utility of high-fidelity Cas9 variants, we produced sgRNAs with matched 5’ nucleotides by using a self-cleaving ribozyme. Thus, each sgRNA was fused to a Hammerhead (HH) ribozyme at its 5’-end [13], which generates mature 20-nucleotide (X_20_) sgRNAs after self-cleavage (Fig. 3a). HH ribozyme-fused sgRNAs with matched 5’ nucleotides (termed HH-X_20_) or the mismatched 5’ guanosine nucleotide (termed HH-gX_19_) were tested in combination with Cas9-WT and high-fidelity Cas9 variants in HeLa cells (Fig. 3b). Use of HH-X_20_ sgRNAs rescued the activity of the two Cas9 variants at all six target sites. Thus, indel frequencies obtained with eCas9-1.1 (64 ± 6%) and Cas9-HF1 (55 ± 7%) using HH-X_20_ sgRNAs were comparable to those obtained with Cas9-WT (69 ± 5% or 70 ± 3%) using HH-X_20_ sgRNAs or HH-gX_19_ sgRNAs, respectively (Fig. 3c and Additional file 1: Table S3a). The ratios of indel frequencies of Cas9 variants in complex with HH-X_20_ sgRNAs relative to that of Cas9-WT had medians of 0.9 for eCas9-1.1 and 0.8 for Cas9-HF1 (Additional file 1: Figure S2, Table S3b). The two Cas9 variants were poorly active when combined with HH-gX_19_ sgRNAs, demonstrating that the rescue of high-fidelity variants was due to matched nucleotides at the 5’ end rather than the ribozyme fusion itself. As expected, Cas9-WT was equally efficient with HH-X_20_, HH-X_19_, and gX_19_ sgRNAs (*P* = 0.36, HH-X_20_ vs HH-X_19_; *P* = 0.28, HH-X_20_ vs gX_19_; *P* = 0.31, HH-X_19_ vs gX_19_) (Fig. 3c and Additional file 1: Table S3a). Editing efficiencies of eCas9-1.1 and Cas9-HF1 were also increased with HH-X_20_ sgRNAs in HEK293T cells (Additional file 1: Figure S3 and Table S4).

### Specificities of Cas9-WT and high-fidelity Cas9 variants in combination with HH-X_20_ sgRNAs

**Fig. 4:**
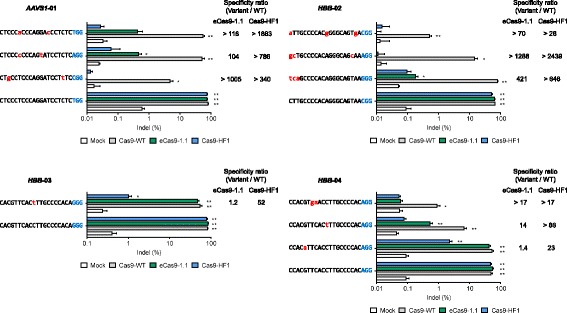
Specificities of high-fidelity Cas9 variants in combination with HH-X_20_ sgRNA. The HH-X_20_ sgRNA expression plasmid was co-transfected with the plasmid encoding Cas9-WT or a Cas9 variant into HeLa cells. Indel frequencies at on- and off-target sites were measured by targeted deep sequencing. PAM sequences are shown in *blue*. Mismatched bases are shown in *red*. The specificity ratio indicates the fold difference between the ratio of on-target indel frequencies to off-target indel frequencies obtained using Cas9 variants and that obtained using Cas9-WT. Error bars, s.e.m. of three biological replicates. Indel frequencies significantly above those of the mock transfected sample are shown by *asterisks* (* *P* < 0.05, ** *P* < 0.01)

Next, we compared the specificities of the two Cas9 variants in complex with HH-X_20_ sgRNAs by measuring mutation frequencies at known off-target sites in HeLa cells. The *CCR5*-01- and *EMX1*-05-specific sgRNAs have no known off-target sites and were excluded from this analysis. At most of the off-target sites that differed from their respective on-target sites by one to three nucleotides, the two Cas9 variants showed much lower indel frequencies than Cas9-WT (Fig. 4). Of note, Cas9-HF1 was able to discriminate against three off-target sites (one *HBB*-03 off-target site and two *HBB*-04 off-target sites), each with a single nucleotide mismatch. These results show that attenuated Cas9 variants retain their high specificities when combined with HH-X_20_ sgRNAs. eCas9-1.1 and Cas9-HF1 also showed high specificities with HH-X_20_ sgRNAs in HEK293T cells (Additional file 1: Figure S4).

## Discussion and conclusions

In summary, we showed here that newly developed, high-specificity Cas9 variants, unlike the wild-type (WT) protein, are often inefficient at target sites with a mismatch at the 5’ terminus, unequivocally demonstrating the contribution of the 5’ nucleotide to the high specificity of CRISPR-Cas9 in human cells for the first time. Of note, a single 5’-end mismatch between the sgRNA and target DNA is largely tolerated by Cas9-WT. The two attenuated Cas9 variants, however, contain several alanine substitutions to weaken ionic interactions between the protein and target DNA, which can make them sensitive to a single mismatch even at the 5’ terminus. By matching the first nucleotide of sgRNAs to target DNA via the self-cleaving activity of a HH-ribozyme fusion, highly specific genome editing was achieved without sacrificing on-target editing efficiency. As an alternative to using a HH-ribozyme fusion, sgRNAs with matched 5’ non-G nucleotides could be created via tRNA fusion [14] or chemical synthesis [15] and combined with the two high-fidelity Cas9 variants. Delivery of pre-assembled Cas9 variant ribonucleoproteins [16] rather than Cas9- and sgRNA-encoding plasmids may further improve genome-wide target specificities of CRISPR genome editing. Our method expands targetable sites for high-specificity Cas9 variants, allowing broad applications in research and medicine.

## Methods

### Construction of high-fidelity Cas9 variant-encoding plasmids and the HH-ribozyme-fused sgRNA-encoding plasmid

eCas9-1.1- and Cas9-HF1-encoding plasmids (p3s-eCas9-1.1, Addgene #104172; p3s-Cas9-HF1, Addgene #104173) were created via site-directed mutagenesis of a WT Cas9 construct (p3s-Cas9-HN, Addgene #104171). HH-ribozyme sgRNA constructs were cloned via ligation of annealed oligonucleotides that included a HH-ribozyme sequence and a protospacer sequence into a plasmid (pRG2, Addgene #104174) in which sgRNA expression is under the control of the U6 promoter.

### Cell culture and transfection

HeLa cells (ATCC, CCL-2) and HEK 293 T/17 cells (ATCC, CRL-11268) were maintained in Dulbecco’s modified Eagle’s medium (DMEM) supplemented with 100 units/mL penicillin, 100 μg/mL streptomycin, 0.1 mM non-essential amino acids, and 10% fetal bovine serum (FBS). 0.8 × 10^5^ HeLa cells and 2 × 10^5^ HEK293T/17 cells were transfected with the Cas9-encoding plasmid (0.5 μg) and sgRNA expression plasmid (0.5 μg) using Lipofectamine 2000 (Invitrogen) according to the manufacturer’s protocol.

#### Western blotting

The Cas9-WT and Cas9 variant proteins expressed in HeLa cells after transfection were detected using western blotting. Cas9 and GAPDH were detected using anti-Cas9 (Abcam, ab191468) and anti-GAPDH (Santa Cruz Biotechnology, sc-32233) primary antibodies. Goat anti-mouse IgG-HRP antibody (Santa Cruz Biotechnology, sc-2005) was used for signal detection. ImageQuant LAS4000 (GE healthcare) was used for digital imaging.

### Targeted deep sequencing

The on-target and off-target regions were polymerase chain reaction (PCR) amplified for NGS library construction. Pooled PCR amplicons were sequenced using MiniSeq with a TruSeq HT Dual Index system (Illumina). Indel frequencies were obtained using Cas-Analyzer [17].

## Additional file

Additional file 1: Figure S1. Comparison of editing efficiencies of Cas9-WT and high-fidelity Cas9 variants using gX19 and gX20 sgRNAs in HEK293T cells. **Figure S2.** Comparison of relative indel frequencies of Cas9 variants normalized to that of Cas9-WT in HeLa cells. **Figure S3.** Comparison of indel frequencies of HH-X_20_, HH-gX_19_, and gX_19_ sgRNAs in combination with Cas9-WT and Cas9 variants in HEK293T cells. **Figure S4.** Specificities of high-fidelity Cas9 variants in combination with HH-X_20_ sgRNA in HEK293T cells. **Table S1.** Indel frequencies of Cas9-WT, eCas9-1.1, and Cas9-HF1 combined with gX_19_ sgRNAs at 26 target sites with an HX_19_ sequence. **Table S2.** Comparison of Cas9-WT and Cas9 variants using gX_19_ sgRNAs at 26 target sites with an HX_19_ sequence. **Table S3.** Comparison of indel frequencies of HH-X_20_, HH-gX_19_, and gX_19_ sgRNAs in combination with Cas9-WT and Cas9 variants in HeLa cells. **Table S4.** Comparison of indel frequencies of HH-X_20_, HH-gX_19_, and gX_19_ sgRNAs in combination with Cas9-WT and Cas9 variants in HEK293T cells. (PDF 497 kb)

## Abbreviations

Cas9-HF1: Cas9 high-fidelity variant 1
Cas9-WT: Wild-type Cas9
DSBs: DNA double strand breaks
eCas9-1.1: Enhanced Cas9-1.1
HH: Hammerhead
Indels: Insertions or deletions
NHEJ: Non-homologous end-joining
sgRNAs: Single guide RNAs
