# Supplementary material for: Rescue of high-specificity Cas9 variants using sgRNAs with matched 5’ nucleotides
Source: Genome Biol. 2017 Nov 15;18:218. doi: 10.1186/s13059-017-1355-3 (PMC5686910; doi:10.1186/s13059-017-1355-3)
Supplement: Additional file 1: Figure S1. — Comparison of editing efficiencies of Cas9-WT and high-fidelity Cas9 variants using gX19 and gX20 sgRNAs in HEK293T cells. Figure S2. Comparison of relative indel frequencies of Cas9 variants normalized to that of Cas9-WT in HeLa cells. Figure S3. Comparison of indel frequencies of HH-X20, HH-gX19, and gX19 sgRNAs in combination with Cas9-WT and Cas9 variants in HEK293T cells. Figure S4. Specificities of high-fidelity Cas9 variants in combination with HH-X20 sgRNA in HEK293T cells. Table S1. Indel frequencies of Cas9-WT, eCas9-1.1, and Cas9-HF1 combined with gX19 sgRNAs at 26 target sites with an HX19 sequence. Table S2. Comparison of Cas9-WT and Cas9 variants using gX19 sgRNAs at 26 target sites with an HX19 sequence. Table S3. Comparison of indel frequencies of HH-X20, HH-gX19, and gX19 sgRNAs in combination with Cas9-WT and Cas9 variants in HeLa cells. Table S4. Comparison of indel frequencies of HH-X20, HH-gX19, and gX19 sgRNAs in combination with Cas9-WT and Cas9 variants in HEK293T cells. (PDF 497 kb) [file 13059_2017_1355_MOESM1_ESM.pdf]

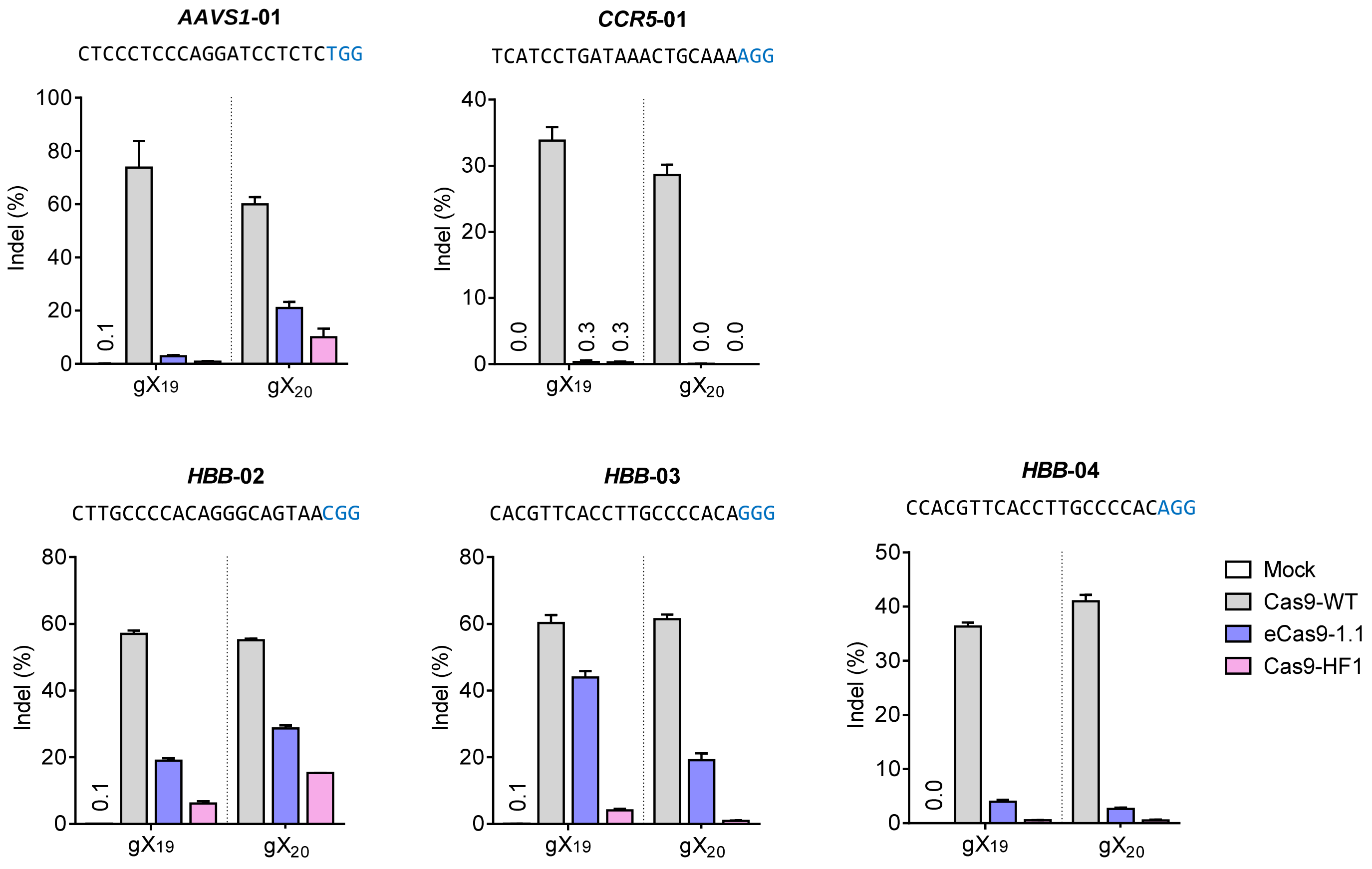

Figure S1. Comparison of editing efficiencies of Cas9-WT and high-fidelity Cas9 variants using gX_{19} and gX_{20} sgRNAs in HEK293T cells.

Indel frequencies (measured by targeted deep sequencing) of Cas9-WT and Cas9 variants at five on-target sites with a 5' C or 5' T nucleotide obtained using gX_{19} and gX_{20} sgRNAs in HEK293T cells. PAM sequences are shown in blue. Error bars, s.e.m. of three biological replicates.

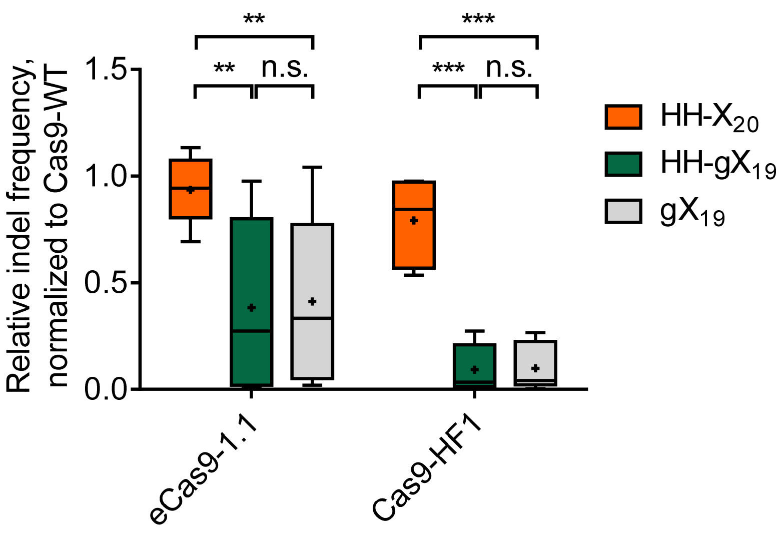

Figure S2. Comparison of relative indel frequencies of Cas9 variants normalized to that of Cas9-WT in HeLa cells.

Distributions of relative indel frequencies of Cas9 variants normalized to that of Cas9-WT obtained using HH-X_{20}, HH-gX_{19} and gX_{19} sgRNAs at six endogenous target sites in HeLa cells. Box and whisker plots: center lines show the medians; crosses show the means. Statistical significances were calculated by paired t-test. ** $P < 0.01$, *** $P < 0.001$.

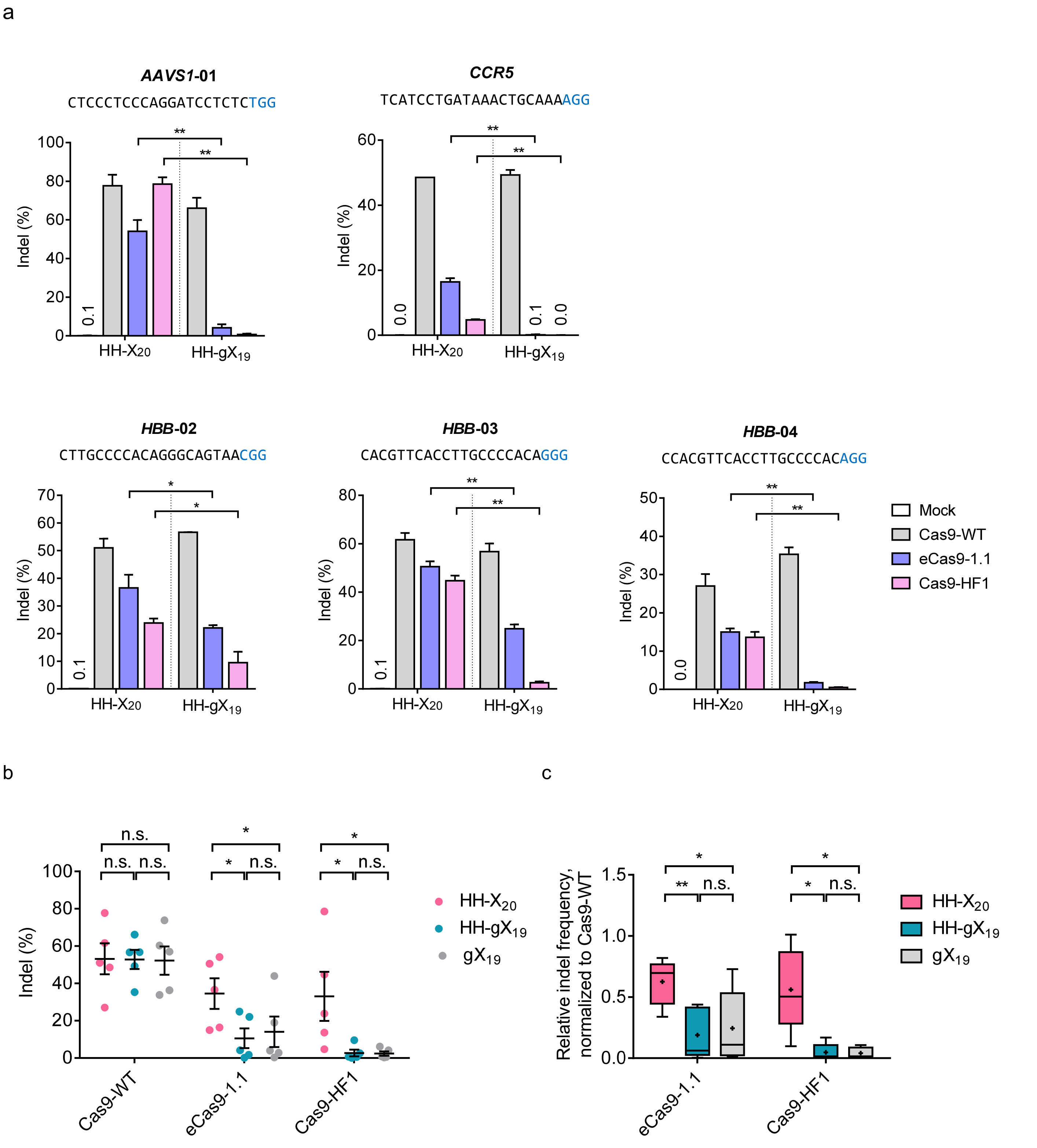

Figure S3. Comparison of indel frequencies of HH-X_{20}, HH-gX_{19} and gX_{19} sgRNAs in combination with Cas9-WT and Cas9 variants in HEK293T cells.

(a) HH-X_{20} or HH-gX_{19} were tested in combination with Cas9-WT and high-fidelity Cas9 variants at five target sites in HEK293T cells. Indel frequencies were measured using targeted deep sequencing. The PAM is shown in blue. Error bars, s.e.m. of three biological replicates. Statistical significances were calculated by unpaired t-test. * $P < 0.05$, ** $P < 0.01$. (b) Mean indel frequencies ± s.e.m. are shown at the five target sites in HEK293T cells. Statistical significances were calculated by paired t-test. * $P < 0.05$. (c) Distributions of relative indel frequencies of eCas9-1.1 and Cas9-HF1 normalized to that of Cas9-WT. Box and whisker plots: center lines show the medians; crosses show the means.

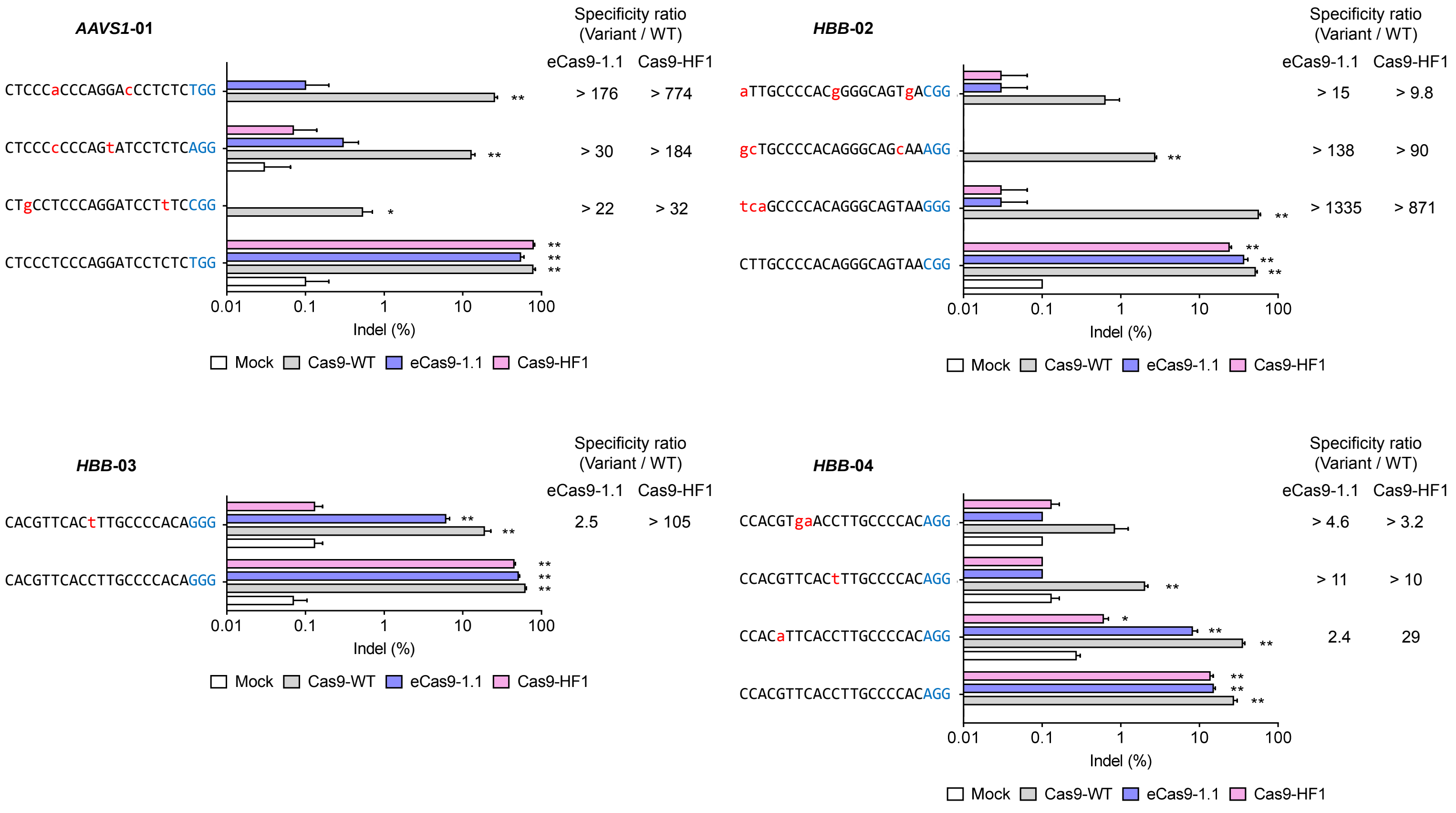

Figure S4. Specificities of high-fidelity Cas9 variants in combination with HH-X_{20} sgRNA in HEK293T cells.

The HH-X_{20} sgRNA expression plasmid was co-transfected with the plasmid encoding Cas9-WT or a Cas9 variant into HEK293T cells. Indel frequencies at on- and off-target sites were measured by targeted deep sequencing. PAM sequences are shown in blue. Mismatched bases are shown in red. The specificity ratio indicates the fold difference between the ratio of on-target indel frequencies to off-target indel frequencies obtained using Cas9 variants and that obtained using Cas9-WT. Error bars, s.e.m. of three biological replicates. Indel frequencies significantly above those of the mock transfected sample are shown by asterisks (* $P < 0.05$, ** $P < 0.01$).

Table S1. Indel frequencies of Cas9-WT, eCas9-1.1 and Cas9-HF1 combined with gX_{19} sgRNAs at 26 target sites with an HX_{19} sequence.

gX_{19} sgRNAs were co-transfected with Cas9-WT or Cas9 variants into HeLa cells and indel frequencies at 26 sites were meausred by targeted deep sequencing. Relative indel frequency is the ratio of indel (%) of Cas9 variant to that of Cas9-WT. Six target sites analyzed in further study with HH-X20 sgRNA are shown in yellow.

				Indel (%)												Relative Indel frequency, normalized to Cas9-WT	
				Mock			Cas9-WT			eCas9-1.1			Cas9-HF1				
gene	sgRNA #	Spacer type	Target sequence (X_{20}-PAM)	Mean (%)	STDEV	N	Mean (%)	STDEV	N	Mean (%)	STDEV	N	Mean (%)	STDEV	N	eCas9-1.1	Cas9-HF1
AAVS1	01	CX_{19}	CTCCCTCCCAGGATCCTCTCTGG	0.59	0.21	3	71.9	8.1	3	6.7	0.3	3	2.7	0.4	3	0.09	0.04
HBB	02	CX_{19}	CTTGCCCCACAGGGCAGTAACGG	0.09	0.07	3	78.2	5.8	3	44.8	7.6	3	16.4	3.1	3	0.57	0.21
HBB	03	CX_{19}	CACGTTCACCTTGCCCCACAGGG	0.40	0.19	3	75.0	6.4	3	78.2	2.4	3	20.0	1.5	3	1.04	0.27
HBB	04	CX_{19}	CCACGTTCACCTTGCCCCACAGG	0.09	0.03	3	48.4	11.6	3	33.0	1.2	3	2.2	0.4	3	0.68	0.05
EMX1	C1	CX_{19}	CGGCAGAAGCTGGAGGAGGAAGG	0.05	0.02	2	50.9	5.8	2	8.0	0.3	2	0.1	0.0	2	0.16	0.00
EMX1	C2	CX_{19}	CACGAAGCAGGCCAATGGGGAGG	0.11	0.08	2	52.1	2.8	2	52.0	5.8	2	19.2	3.6	2	1.00	0.37
EMX1	C3	CX_{19}	CGATGTCACCTCCAATGACTAGG	0.00	0.00	2	69.5	12.6	2	29.9	9.6	2	2.2	0.3	2	0.43	0.03
AAVS1	T1	TX_{19}	TGCTTACGATGGAGCCAGAGAGG	0.01	0.01	2	66.1	9.8	2	0.0	0.0	2	0.0	0.0	2	0.00	0.00
AAVS1	T2	TX_{19}	TAAGCAAACCTTAGAGGTTCTGG	0.00	0.00	2	75.0	6.9	2	10.0	2.9	2	6.2	0.6	2	0.13	0.08
AAVS1	T3	TX_{19}	TCTCCTTGCCAGAACCTCTAAGG	0.01	0.01	2	52.6	11.2	2	35.2	17.7	2	10.9	9.1	2	0.67	0.21
HBB	T1	TX_{19}	TCTGCCGTTACTGCCCTGTGGGG	0.00	0.00	2	77.8	0.1	2	75.8	4.5	2	70.4	3.5	2	0.97	0.90
HBB	T2	TX_{19}	TGGTCTACCCTTGGACCCAGAGG	0.02	0.02	2	39.1	16.3	2	30.2	18.0	2	32.4	13.1	2	0.77	0.83
HBB	T3	TX_{19}	TCAAAGAACCTCTGGGTCCAAGG	0.02	0.03	2	46.9	15.9	2	0.1	0.1	2	0.0	0.0	2	0.00	0.00
HBB	T4	TX_{19}	TCCACTCCTGATGCTGTTATGGG	0.00	0.00	2	46.8	1.2	2	1.1	0.4	2	0.4	0.1	2	0.02	0.01
EMX1	04	TX_{19}	TCTGGAACCACACCTTCACCTGG	0.01	0.01	3	81.5	1.6	3	80.3	4.2	3	81.5	4.0	3	0.99	1.00
EMX1	05	TX_{19}	TGTACTTTGTCCTCCGGTTCTGG	0.01	0.01	3	84.8	7.9	3	5.1	0.5	3	2.3	0.5	3	0.06	0.03
CCR5	01	TX_{19}	TCATCCTGATAAACTGCAAAAGG	0.10	0.05	3	74.1	5.1	3	1.4	0.6	3	0.2	0.1	3	0.02	0.00
AAVS1	A1	AX_{19}	AGATAAGGAATCTGCCTAACAGG	0.01	0.01	2	73.0	0.0	2	71.9	9.1	2	64.2	12.2	2	0.98	0.88
AAVS1	A2	AX_{19}	AGACCCAATATCAGGAGACTAGG	0.02	0.03	2	76.6	3.5	2	80.2	5.7	2	80.5	1.9	2	1.05	1.05
HBB	A1	AX_{19}	AAGGTGAACGTGGATGAAGTTGG	0.00	0.00	2	69.9	4.3	2	63.6	1.8	2	65.1	8.4	2	0.91	0.93
EMX1	01	AX_{19}	ACCGGAGGACAAAGTACAAACGG	0.02	0.02	3	87.6	0.6	3	88.3	0.8	3	88.9	2.3	3	1.01	1.01
EMX1	02	AX_{19}	ATTGCCACGAAGCAGGCCAATGG	0.01	0.02	3	83.4	0.8	3	81.9	1.4	3	84.9	4.2	3	0.98	1.02
EMX1	03	AX_{19}	ACCACACCTTCACCTGGGCCAGG	0.01	0.00	3	80.9	3.3	3	81.1	2.0	3	84.5	3.7	3	1.00	1.04
CCR5	13	AX_{19}	AAAGATAGTCATCTTGGGGCTGG	0.01	0.01	3	77.2	2.8	3	84.0	2.0	3	83.6	0.9	3	1.09	1.08
CCR5	16	AX_{19}	AAGTGTGATCACTTGGGTGGTGG	0.04	0.03	3	87.4	2.5	3	88.7	1.0	3	87.4	2.3	3	1.01	1.00
CCR5	18	AX_{19}	AGATGACTATCTTTAATGTCTGG	0.00	0.00	3	86.3	3.3	3	84.6	0.8	3	75.5	5.3	3	0.98	0.88
EMX1	N/A	GX_{19}	GAGTCCGAGCAGAAGAAGAAGGG	0.39	0.03	3	91.9	1.1	3	95.8	3.0	3	93.1	0.9	3	1.04	1.01
VEGFA	03	GX_{19}	GGTGAGTGAGTGTGTGCGTGTGG	0.01	0.01	3	95.4	1.9	3	95.9	1.6	3	94.3	2.1	3	1.01	0.99

Table S2. Comparison of Cas9-WT and Cas9 variants using gX_{19} sgRNAs at 26 target sites with an HX_{19} sequence.

Means of indel (%) plotted in Figure 2a and means and medians of relative indel frequency of Cas9 variant to that of Cas9-WT at 26 sites plotted in Figure 2b.

	Indel (%)									Relative Indel frequency, normalized to Cas9-WT			
	Cas9-WT			eCas9-1.1			Cas9-HF1			eCas9-1.1		Cas9-HF1	
Spacer type	Mean (%)	S.E.M	N	Mean (%)	S.E.M	N	Mean (%)	S.E.M	N	Mean	Median	Mean	Median
CX_{19}	63.7	4.8	7	36.1	9.5	7	9.0	3.4	7	0.57	0.57	0.14	0.05
TX_{19}	64.5	5.3	10	23.9	9.9	10	20.4	9.8	10	0.36	0.10	0.31	0.06
AX_{19}	80.3	2.2	9	80.5	2.7	9	79.4	3.1	9	1.00	1.00	0.99	1.01

Table S3. Comparison of Indel frequencies of HH-X_{20}, HH-gX_{19} and gX_{19} sgRNAs in combination with Cas9-WT and Cas9 variants in HeLa cells

Indel frequencies (a) and relative indel frequencies of Cas9 variants normalized to that of Cas9-WT (b) at six target sites in HeLa cells.

a

	Indel (%)								
	Cas9-WT			eCas9-1.1			Cas9-HF1		
gene	HH-X_{20}	HH-gX_{19}	gX_{19}	HH-X_{20}	HH-gX_{19}	gX_{19}	HH-X_{20}	HH-gX_{19}	gX_{19}
AAVS1-01	80.4	67.6	71.9	74.4	7.1	6.7	73.6	1.7	2.7
HBB-02	67.3	70.4	78.2	64.7	52.1	44.8	52.0	19.3	16.4
HBB-03	81.2	79.3	75.0	85.7	77.3	78.2	78.9	14.9	20.0
HBB-04	49.5	58.2	48.4	56.1	25.8	33.0	48.3	2.4	2.2
CCR5-01	75.4	73.7	74.1	63.5	1.0	1.4	40.4	0.2	0.2
EMX1-05	59.5	72.8	84.8	41.2	1.6	5.1	34.5	1.2	2.3
Median	71.4	71.6	74.6	64.1	16.4	19.8	50.2	2.1	2.5
Mean	68.9	70.3	72.1	64.3	27.5	28.2	54.6	6.6	7.3

b

	Relative Indel frequency, normalized to Cas9 WT					
	eCas9-1.1			Cas9-HF1		
gene	HH-X_{20}	HH-gX_{19}	gX_{19}	HH-X_{20}	HH-gX_{19}	gX_{19}
AAVS1-01	0.93	0.10	0.09	0.92	0.03	0.04
HBB-02	0.96	0.74	0.57	0.77	0.27	0.21
HBB-03	1.06	0.98	1.04	0.97	0.19	0.27
HBB-04	1.13	0.44	0.68	0.98	0.04	0.05
CCR5-01	0.84	0.01	0.02	0.54	0.00	0.00
EMX1-05	0.69	0.02	0.06	0.58	0.02	0.03
Median	0.94	0.27	0.33	0.84	0.03	0.04
Mean	0.94	0.38	0.41	0.79	0.09	0.10

Table S4. Comparison of Indel frequencies of HH-X_{20}, HH-gX_{19} and gX_{19} sgRNAs in combination with Cas9-WT and Cas9 variants in HEK293T cells

Indel frequencies (a) and relative indel frequencies of Cas9 variants normalized to that of Cas9-WT (b) at five target sites in HEK293T cells.

a

	Indel (%)								
	Cas9-WT			eCas9-1.1			Cas9-HF1		
gene	HH-X_{20}	HH-gX_{19}	gX_{19}	HH-X_{20}	HH-gX_{19}	gX_{19}	HH-X_{20}	HH-gX_{19}	gX_{19}
AAVS1-01	77.7	66.1	73.8	54.1	4.1	2.8	78.5	0.7	0.8
HBB-02	51.0	56.6	57.0	36.5	22.1	19.0	23.8	9.5	6.1
HBB-03	61.6	56.7	60.3	50.5	24.9	43.9	44.7	2.6	4.1
HBB-04	27.0	35.3	36.3	15.0	1.7	4.0	13.6	0.5	0.5
CCR5-01	48.5	49.3	33.8	16.4	0.1	0.3	4.7	0.0	0.3
Median	51.0	56.6	57.0	36.5	4.1	4.0	23.8	0.7	0.8
Mean	53.2	52.8	52.2	34.5	10.6	14.0	33.1	2.7	2.4

b

	Relative Indel frequency, normalized to Cas9 WT					
	eCas9-1.1			Cas9-HF1		
gene	HH-X_{20}	HH-gX_{19}	gX_{19}	HH-X_{20}	HH-gX_{19}	gX_{19}
AAVS1-01	0.70	0.06	0.04	1.01	0.01	0.01
HBB-02	0.72	0.39	0.33	0.47	0.17	0.11
HBB-03	0.82	0.44	0.73	0.73	0.05	0.07
HBB-04	0.55	0.05	0.11	0.50	0.01	0.01
CCR5-01	0.34	0.00	0.01	0.10	0.00	0.01
Median	0.70	0.06	0.11	0.50	0.01	0.01
Mean	0.62	0.19	0.24	0.56	0.05	0.04
